# Exploring the role of DNA damage response in seed priming to uncover key players for multi-stress tolerance

**DOI:** 10.1093/jxb/eraf237

**Published:** 2025-05-29

**Authors:** Anca Macovei, Andrea Pagano, Conrado Duenas, Susana Araujo, Alma Balestrazzi

**Affiliations:** Department of Biology and Biotechnology ‘L. Spallanzani’, University of Pavia, Pavia, Italy; Department of Biology and Biotechnology ‘L. Spallanzani’, University of Pavia, Pavia, Italy; Department of Biology and Biotechnology ‘L. Spallanzani’, University of Pavia, Pavia, Italy; Laboratório Colaborativo Montanhas de Investigação (MORE), Bragança, Portugal; Centro de Investigação da Montanha (CIMO), LA SusTEC, Instituto Politécnico de Bragança, Bragança, Portugal; Department of Biology and Biotechnology ‘L. Spallanzani’, University of Pavia, Pavia, Italy; University of Szeged, Hungary

**Keywords:** DNA damage response, germination, multi-factorial stress combination, pre-germinative metabolism, seed priming

## Abstract

Combined climatic stressors result in cumulative damage and unpredictable shocks to seed systems. Seed priming, a pre-sowing technique used to enhance seed vigour, is a key tool to face climate change. Priming agents boost the DNA damage response during early seed imbibition, preserving genome integrity and ensuring germination. Based on these premises, to what extent could the DNA damage response support the seed response to multiple stresses? How could seed priming enhance DNA repair to better fight combined stressors? How far are we from understanding the mechanisms for multiple stress tolerance that can contribute to improved resilience in seeds? The state of the art is critically discussed considering the scanty knowledge on this topic, highlighting the complex scenario of multi-factorial stress combinations. Case studies showing the efficacy of seed priming in promoting multiple stress response are reviewed, integrating the role of cross-stress tolerance, target germplasm (from major to orphan crops), and the contribution of advanced omics/phenotyping tools. Expanding current knowledge in seed biology, by focusing on the impact of multiple climatic stressors, is a challenge since there are still relevant open questions concerning the way in which the DNA damage response can be utilized in seeds that must be addressed.

## Teaser

We highlight the potentialities of harnessing the seed DNA damage response throughout priming to obtain new climate-ready crops.

## Introduction

The global climate crisis, resulting from recurrent extreme events (e.g. heat waves, cold waves, droughts, wildfires, floods, heavy rain, and storms), poses risks to food security and health, enhancing the competition for resources, while conflicts and political instability further exasperate this complex context (Cattaneo and Foreman, 2023). Under climate change conditions, a wide range of abiotic and biotic stressors act in combination, resulting in cumulative damage and unpredictable shocks to food systems and ecosystem integrity (Sparling *et al*., 2024). Stressors are defined as external signals that, depending on the extent and intensity of exposure, might severely impact crop growth, triggering relevant changes in metabolic pathways and physiological responses. Temperature fluctuations, as climatic stressors, trigger frost damage or heat injury, affecting the vegetative and reproductive stage (Guo *et al*, 2024; Lai *et al*., 2024), whereas waterlogging and flooding cause saturated soil conditions leading to oxygen deficiency (Manghwar *et al*., 2024). Other stressors alter the soil and plant water status, promoting drought and salt toxicity (Pascolini-Campbell, 2022), and affect plant nutrition as in the case of nitrogen limitation (Seufert *et al*., 2019). The impact of single climatic stressors on crop agronomic performance has been extensively investigated, from bench to field, at the molecular and physiological level; however, only a few models depicting the plant response to multiple stressors [also defined as multi-factorial stress combination (MFSC)] are currently available (Rötter *et al*., 2018; Webber *et al*., 2022), and the literature concerning the impact on seed quality and germination performance is still scanty.

Seed vigour, a complex agronomic trait, influenced by genetic and environmental factors, encompassing seed longevity, germination performance, seedling development, and stress tolerance, is severely challenged by climatic stressors. Heat stress affects seed development, impacting seed size and number, resulting in overall poor seed vigour (Sehgal *et al*., 2018; Devi *et al*., 2023). Variability in temperature patterns can trigger seed dormancy (Klupczyńska and Pawłowski, 2021; Reed *et al*., 2022), and deleterious effects on seed quality are observed under storage, exacerbating the drawbacks of ageing (Bizouerne *et al*., 2023). High-quality seeds are endowed with protective mechanisms that allow them to repair damage to nucleic acids, proteins, and lipid membranes (Pagano *et al*., 2017; Dirk and Downie, 2018; Doria *et al*., 2019; Waterworth *et al*., 2019, 2024), thus favouring adaptation to harsh environments. Such capacities are continuously challenged by climate change (Bailly and Gomez-Roldan, 2023). Genes, proteins, and metabolites involved in protective mechanisms, ranging from redox maintenance, free radical scavenging, membrane and DNA damage repair, as well as genes ruling seed dormancy and longevity, are envisaged as tools for assessing seed quality, stress tolerance, and the potential contribution to climate change resilience (Corbineau *et al*., 2023; Pagano *et al*., 2023; Sincinelli *et al*., 2025). The seed responsiveness to signalling molecules, such as the antagonist phytohormones abscisic acid (ABA) and gibberellin (Xian *et al*., 2024), as well as ethylene (Corbineau, 2024) and brassinosteroid (Kim *et al*., 2019), is fundamental to shape germination. A key determinant of seed germination is redox control based on the crosstalk between ROS (reactive oxygen species), NO (nitric oxide), and the seed antioxidant machinery (Bailly, 2019; Bailly and Gomez-Roldan, 2023). In the context of seed pre-germinative metabolism, additional levels of interaction have been reported for free radical- and phytohormone-based molecular networks (Jhanji *et al*., 2024). The seed non-enzymatic antioxidants, key protective agents against oxidative damage, can also act in signalling pathways as in the case of ascorbate (Niu *et al*., 2024). The iron–sulfur (Fe–S) clusters, essential cofactors in redox reactions, are used to sense the intracellular environment, and to transmit an iron-mediated signal that can affect the stability of the target proteins. Most of the Fe–S cluster proteins participate in the modulation of processes, such as DNA repair, required for successful germination (Novoa-Aponte *et al*., 2024).

Among the plethora of molecular events triggered when water uptake by the quiescent seed starts, the DNA damage response (DDR) stands as an essential component required to preserve genome integrity of embryo cells, paving the way to successful germination (Waterworth *et al*., 2019, 2022; Pagano *et al*., 2023; Waterworth *et al*., 2024; Sincinelli *et al*., 2025). The contribution of DDR to the ability of the seed to withstand adverse environments has been reported in relation to different abiotic stresses, but knowledge concerning multiple stress tolerance is still scant. The present review proposes to critically address these issues, in view of the current state of the art.

## Multi-factorial stress combination: complexity and challenges

### Definition and models

The framework proposed by Webber *et al*. (2022) is based on four stressor combinations. ‘Single exposure’ refers to the combination of multiple climatic stressors or soil conditions, such as the concomitant occurrence of soil water deficit and soil salinity. The latter triggers an osmotic effect that further decreases water availability, enhancing cellular damage. Such a scenario has been analysed using simulation models able to monitor the effects of climate and soils on crop management (Webber *et al*., 2022). In the case of ‘no direct interaction’, exposure to different stressors does not cause interactions between different metabolic processes, since each stressor acts independently. This has been reported for the combination of heat and nitrogen stressors characterized by distinct targets (grain production and leaf expansion, respectively), using the SIMPLACE (Scientific Impact assessment and Modeling Platform for Advanced Crop and Ecosystem management) modelling framework (Gaiser *et al*., 2013; Webber *et al*., 2015). When a ‘known interaction’ is present, exposure to multiple stressors addresses the same metabolic processes, resulting in synergistic or antagonistic responses. This has been described for the combination of heat and drought stress that leads to extremely severe damage, compared with that observed when separate stresses are applied (Suzuki *et al*., 2014). Finally, multiple stressors result in ‘unknown interaction’ when the related mechanisms are poorly understood, such as for the combination of ozone and drought stress, frost and salinity, and require in-depth experimental investigations to provide data for modelling (Webber *et al*., 2022).

### Impact of MFSC on crop physiology and productivity

The impact of multiple stresses on crop physiology and productivity has been assessed in a wide range of species, under both controlled and field conditions (Zandalinas and Mittler, 2022; Da Ros *et al*., 2023; Peláez-Vico *et al*., 2024; Sinha *et al*., 2024). When exposed to multiple stressors, plants can adopt different protective strategies, using only one or a combination of different stress responses, or eventually triggering novel mechanisms (Joshi *et al*., 2024). The complexity of MFSC is a challenge that delays the ongoing efforts made to investigate and decipher the key processes and players contributing to multi-stress tolerance, required to build strong climate resilience profiles (Jing *et al*., 2024). Studies performed in both model and crop plants have highlighted distinctive molecular features, such as the enrichment of antioxidant components involved in ROS scavenging, and Fe–S metabolism detected in *Arabidopsis thaliana* (L.) Heynh, rice (*Oryza sativa* L.), and maize (*Zea mays* L.) (Zandalinas and Mittler, 2022; Sinha *et al*., 2024). The common liverwort *Marchantia polymorpha* L., a model plant with a simplified life cycle and regulatory networks, has been tested using seven abiotic stresses (darkness, high light, cold, heat, nitrogen deficiency, salt, and mannitol) alone and in 19 pairwise combinations, and the resulting impact has been dissected using transcriptomics (Tan *et al*., 2023). According to this study, gene expression patterns under combined stresses can be predicted with mathematic tools (e.g. a simple linear regression; Tan *et al*., 2023). Comparison with the model plant Arabidopsis revealed the degree of conservation in terms of genes involved in abiotic stress response; however, the regression models generated in this study suggest the presence of unknown factors contributing to the integration of multiple stress responses (Tan *et al*., 2023). There is also emerging evidence of the active role of plant microbiomes in the response to MFSC, as well as the ability of the plant to recruit beneficial microorganisms (Beltrán-García *et al*., 2024). The dynamic modulation of the plant microbiome in response to environmental changes has been reported, particularly as a consequence of the active cooperation between microrganisms (Bakker *et al*., 2018). The impact of plant–microbiome interactions in response to multiple stress combinations has been investigated by Tchakounte *et al*. (2020) who tested the ability of selected *Arthrobacter* and *Bacillus* strains to improve the growth performance of tomato plants under simultaneous phosphorus and salt stresses. Bilal *et al*. (2020) were able to enhance soybean (*Glycine max* L.) development under the simultaneous exposure to heavy metals, high temperature, and drought stresses upon inoculation with the multi-abiotic stress-tolerant *Paecilomyces formosus* LHL10 and *Penicillium funiculosum* LHL06 strains. However, the reports in this field are still limited. Open questions concern the way in which plants can influence the microbial communities under multiple stress combinations, as well as the compatibility of a specific microbiota across different crop species and stress combinations. The development of multiple stress-tolerant beneficial microbiota for sustainable agriculture will require in-depth studies, based on metagenomics and metatranscriptomics, and the characterization of the spatio-temporal responses resulting from the plant–microorganism interactions (Ali *et al*., 2023). Although the state of the art is continuously expanding, there are still consistent gaps of knowledge, particularly as concerns the impact of MFSC on seed germination.

### How does MFSC apply to seeds?

Efficient and uniform field establishment is a key requisite for climate-ready crops to maintain productivity under variable environmental conditions (Finch-Savage and Bassel, 2016; Balestrazzi *et al*., 2024). DDR is one of the aspects of seed quality and stress tolerance that still deserves attention, due to its potential in supporting the response to MFSC. In the present review, the state of the art will be presented and critically discussed taking into consideration the current, scanty knowledge on this topic, highlighting the multi-level, complex scenario that accompanies the study of MFSC on seed germination. Climate change severely threatens seed productivity and the agrifood industry; however, negative impacts can be mitigated by seed priming, a pre-sowing technique used to boost germination performance and seedling stress tolerance (Pagano *et al*., 2023). The main features of seed priming are described in Box 1. Successful priming treatments rely on the ability to properly target key players involved in seed pre-germinative metabolism, such as the antioxidant response and DDR, responsible for safeguarding genome integrity, required for successful germination (Waterworth *et al*., 2019, 2022; Pagano *et al*., 2023). Seed priming features two key steps, controlled imbibition and dry-back, in which seeds undergo controlled water uptake to boost seed metabolism and hasten germination, followed by dehydration that brings the seed back to its original moisture content, ready to be sown (Box 1). It is fundamental to stop controlled imbibition before radicle protrusion occurs, otherwise primed seeds will lose desiccation tolerance and they will not survive the dry-back step (Soeda *et al*., 2005). The concept of the rehydration–dehydration cycle is used to represent a standard priming protocol (controlled seed imbibition followed by desiccation or dry-back), providing an experimental context suitable to address some critical issues of the technique, such as those related to imbibition damage and loss and gain of desiccation tolerance (Fabrissin *et al*., 2021; Pagano *et al*., 2022a, b, 2023). Major drawbacks of seed priming also include the reduced storability of primed seeds and the intra- and inter-specific variability in the responses to priming (Pagano *et al*., 2023). While the versatility of seed priming makes it suitable for on-farm administration as well as tailored optimization in industrial contexts, the development of comprehensive models of seed metabolism and the identification of hallmarks of stress tolerance and priming effectiveness can boost the optimization of novel priming techniques focused on multi-stress tolerance (Box 1).

Box 1. **Overview of seed priming technology and models, including its drawbacks and implications of the DNA damage response**

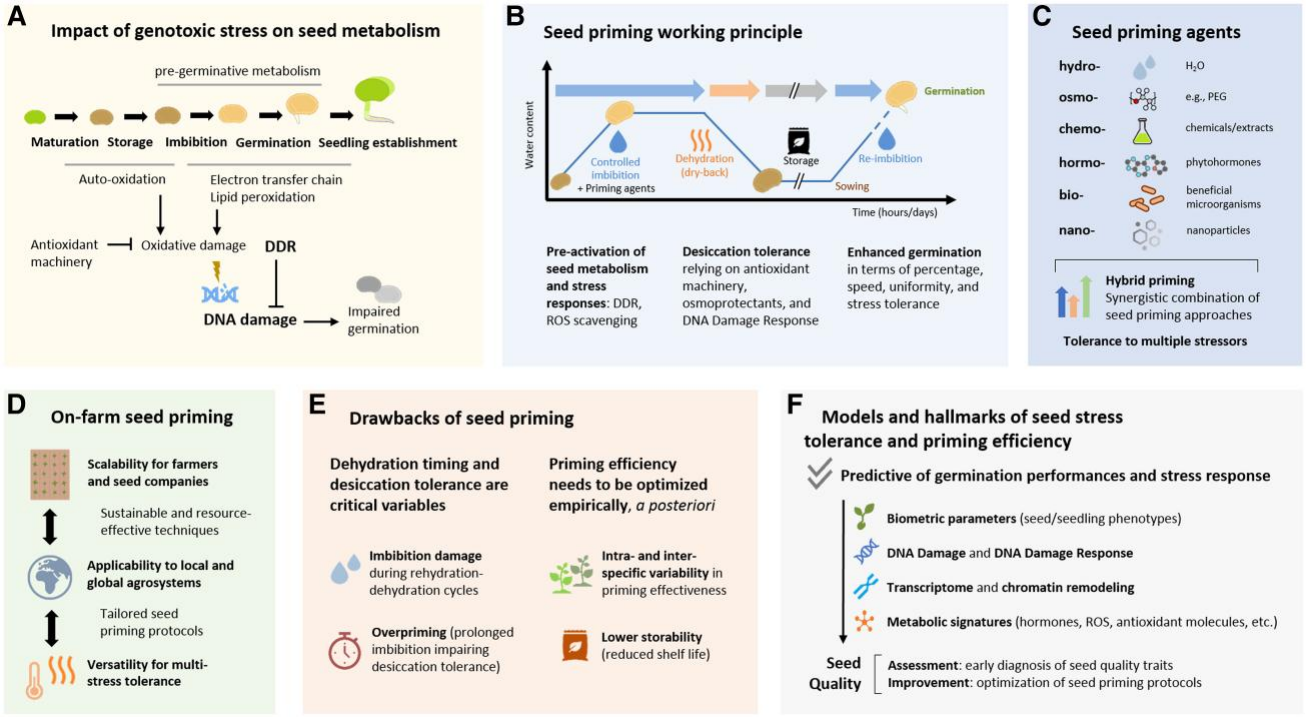

1. Impact of genotoxic stress on seed metabolism. DNA damage can take place as a result of oxidative stress occurring at different stages of seed development, maturation, storage, pre-germinative metabolism, and seedling growth. Whereas oxidative processes can occur non-enzymatically when seed metabolism is inactive under dry storage, metabolic reactivation during imbibition and germination physiologically induces ROS production and potentially threatens DNA integrity and germination performance. The enzymatic and non-enzymatic antioxidant machinery maintains oxidative homeostasis within physiological ranges, while the activation of DNA damage response (DDR) in pre-germinative metabolism repairs the DNA damage that is accumulated during storage (Waterworth *et al*., 2019, 2022; Pagano *et al*., 2023).
2. Working principle of seed priming. Seed priming approaches rely on rehydration–dehydration cycles (an incomplete imbibition followed by a dry-back step) to pre-activate seed metabolism, preparing the seed for a faster and more synchronized germination. Supplementation with controlled stress or specific priming agents (including salts, osmotic agents, thermal treatments, phytohormones, and others) can induce targeted responses, including the enhancement of stress response mechanisms (Pagano *et al*., 2023).
3. Seed priming agents. An array of biological, chemical, and physical agents can be administered in the context of seed priming protocols to induce specific responses in pre-germinative metabolism. Priming agents can be administered alone or in combination so that the resulting hybrid priming protocol induces targeted responses, including tolerance to multiple stressors (Pagano *et al*., 2023; Goufa *et al*., 2025
  ).
4. On-farm seed priming. Seed priming represents a versatile and environmentally friendly solution to enhance germination and field establishment, with protocols that can be tailored on the possibilities and requirements of farmers and seed companies to leverage the potential of seed material in different agro-economical contexts with variable resource availability. The simplest priming technique (hydropriming) has been historically applied and represents a cost-effective approach for farmers compared with the supplementation of priming agents or other pre-sowing vigorization approaches carried out by seed companies (Pagano *et al*., 2023).
5. Drawbacks of seed priming. The main drawbacks of seed priming include the variability in the observed responses among species, varieties, and seed lots. Rehydration and dehydration timing are critical variables since both imbibition and desiccation can induce damage, overcoming the enhancement of germination. Priming applicability is restricted to orthodox seeds that can withstand dehydration steps thanks to the accumulation of LEA (late embryogenesis abundant) proteins and other compounds protecting cell structures and macromolecules in anhydrous environments. In this sense, prolonged imbibition steps can result in the loss of seed desiccation tolerance also in orthodox seeds, impairing seed viability, a condition known as ‘overpriming’. Moreover, the activation of seed metabolism during priming reduces seed longevity after storage, representing another constraint of the technique. The co-occurrence of such drawbacks is variable and difficult to predict, so that priming protocols normally require empirical optimization (Bradford *et al*., 1990; Soeda *et al*., 2005; Fabrissin *et al*., 2021; Pagano *et al*., 2022a, b, 2023).
6. Models and hallmarks of stress tolerance and priming efficiency. Refining the current models of seed metabolism responding to different environmental stressors is a promising route to facilitate the development and optimization of priming protocols toward climate-resilient crops. The identification of biometric traits that are predictive of stress tolerance, including patterns of gene expression, metabolite accumulation profiles, DNA damage and repair dynamics, and chromatin modifications, as hallmarks for seed quality complements the need for empirical validation, facilitating seed quality testing and improvement. Seed quality hallmarks referring to DDR and antioxidant response are useful to decode multi-stress tolerance mechanisms, further expanding their applicative potential for seed quality enhancement (Chen *et al*., 2019; Sharma *et al*., 2022; Pagano *et al*., 2023).

Expanding the current knowledge in seed biology, focusing on the impact of multiple climatic stressors, has become a priority as well as a challenge for the scientific community. The several relevant research questions, still open, listed in Box 2 define the overall scope and rationale that should guide researchers towards a better understanding of how seeds manage MFSC.

Box 2. **List of the main open research questions that need to be addressed to speed up the gain in knowledge on the basic mechanisms ruling the response to multiple climatic stressors in germinating seeds**

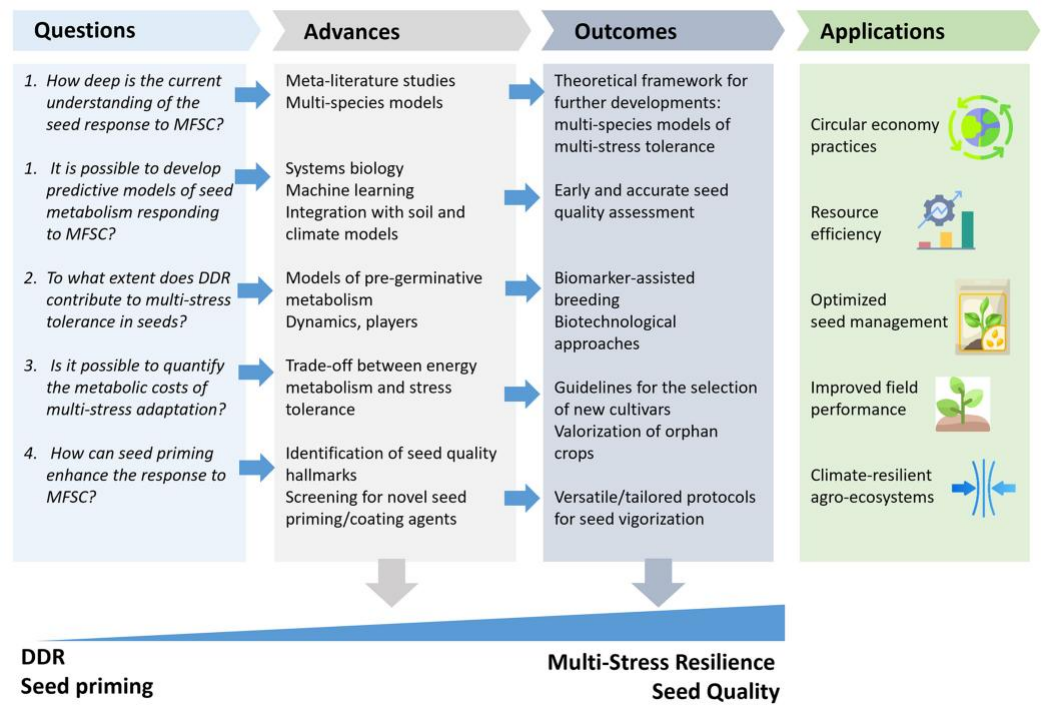

## Impact of multi-factorial stress combination on seed germination

### Current background and main focus

The effects of combined climatic stressors on seed germination have been poorly investigated, however the number of reports addressing this issue is increasing. Most of the studies focus on the impact of high CO_2_ levels on germination, when combined with other abiotic stresses, particularly drought and salinity (Debouza *et al*., 2021), or the effects of high CO_2_ exposure on seed production, biomass, and quality in a range of different crop and wild species (Jablonski *et al*., 2002). The tested species showed an increase in seed number and total seed mass (up to 16% and 4%, respectively), and exposure to high CO_2_ levels did not significantly impact on the reproductive effort of crop species, but for wild species the reproduction performance was limited by 14%. In this case, a consistent carbon allocation was supplied to other structural, defensive, non-reproductive functions (Jablonski *et al*., 2002). Besides such an assessment, interactions between drought, high temperature, and high CO_2_ levels were reported to negatively impact on Arabidopsis germination performance, with additional variability brought by different genotypes (Abo Gamar and Qaderi, 2019). High CO_2_ contributes to global warming that, in turns, accelerates the rise of sea level and indirectly leads to soil salinity along coasts, compromising germination (Dasgupta *et al*., 2015). Similarly, association of high CO_2_ levels with enhanced atmospheric temperature triggers heatwaves and drought conditions that affect germination (Roy *et al*., 2016). The interaction between high CO_2_ and temperature and their influence on seed production was investigated by Hampton *et al*. (2013). The concomitant occurrence of the two stressors resulted in antagonistic effects since the acceleration in seed development and maturation promoted by elevated CO_2_ had to cope with a limited duration of the seed-filling phase and dry matter accumulation. This specific scenario focuses the attention on the way in which effective compensation strategies could be designed, such as through the dissection of the most stress-sensitive time windows for seed development and the type of interactions among stressors such as the exposure time. Action plans to minimize the climate change-associated risks for large-scale seed production could be minimized by changing the location and elevation of production sites, and modifying the sowing date to allow seed filling at optimal temperatures (Hampton *et al*., 2013). The screening for genotypes carrying seed quality traits linked to climate-resilient germination performances is currently ongoing, using advanced molecular tools integrated with agronomic disciplines. The valorization of existing tolerant germplasm is a fundamental step towards the breeding of new climate-ready cultivars. This has been analysed in two soybean cultivars whose harvest index dropped by 70% upon simultaneous exposure to both stresses. Other studies, performed to assess the combined effect of a range of elevated temperature under high CO_2_ levels on seed yield and quality, allowed identification of the optimal temperature for proper seed filling and define the changes affecting major seed components (Thenveettil *et al*., 2024; Vanaja *et al*., 2024).

### Combined abiotic stresses affect grain quality in cereals: insights into basic mechanisms

The expected enhancement in global temperatures, together with the increased frequency of extreme events and other detrimental conditions, will act against food security, fostering deleterious effects such as deterioration of grain quality in cereals (Bentley *et al*., 2022; Zahra *et al*., 2022). It has been reported that the concomitant exposure of rice to drought and heat stress negatively influences the qualitative and quantitative features of seed components; for example, starch metabolism is inhibited due to thermal denaturation of enzymes whereas protein and mineral content is increased (Pravallika *et al*., 2020; Ben Mariem *et al*., 2021). Complex regulatory networks feature multi-level defences ruled by key master regulators such as the *OsRab7* (*ras* gene from rat brain) gene whose overexpression in rice resulted in improved grain yield under combined drought and heat stress (He *et al*., 2018; El-Esawi and Alayafi, 2019). The RAB7 GTPases, involved in the control of endocytic, biosynthetic, and autophagic traffic to the vacuole, contribute to the plant stress response (Rodriguez-Furlan *et al*., 2023); however, additional investigation will be required to better assess its role in the context of seed development and maturation. The search for rice cultivars tolerant to combined heat and drought stress has so far led to the identification of only one tolerant rice cultivar, Nagina 22 (Reshma *et al*., 2021). Combining high CO_2_ and temperature levels compromises early grain filling, seed set, and seed quality in rice, as evidenced by Chaturvedi *et al*. (2017) who compared the response of the heat stress-tolerant NL-44 and high-yield Pusa 1121 cultivars. However, genotype-dependent responses were recorded. Pusa 1121 showed a positive response under elevated CO_2_, while a significant reduction in seed set and sink starch metabolism enzymatic activity was observed under combined stresses. In contrast, NL-44 proved tolerant to elevated CO_2_ and heat stress, resulting in significantly high seed set and active starch metabolism enzymes (Chaturvedi *et al*., 2017). These studies highlight the relevance of germplasm screening as a fruitful approach to identify climate-ready rice cultivars that could be used as donors of multiple stress tolerance in breeding programmes aimed to improve global food productivity under CO_2_-enriched environments. As expected, the search for multiple stress tolerance becomes more challenging when the complexity of multiple stress interactions increases. Combinations of drought, salinity, and high temperature have been explored in rice by Ali *et al*. (2022). Twenty varieties, tolerant to each individual stress, were screened for the presence of SSR (simple sequence repeat) markers link to drought, salinity, and heat stress tolerance, and then exposed to combined stress conditions. Among the tested genotypes, PTB-7 and Nagina 22 proved tolerant to combined drought–salinity and high temperature–salinity treatments, respectively, however they did not show seed germination when the three stresses were applied simultaneously (Ali *et al*., 2022). Due to poor availability of water resources and inadequate irrigation facilities, concomitant salinity and drought stresses frequently affect rice production in saline soils. Wei *et al*. (2023) evaluated the individual and combined effects of salinity and drought on the rice cultivars Nanjing 9108 and Wuyunjing 30, classified as tolerant and susceptible to salt stress, respectively. A significant reduction in grain yield, represented by low panicle length and weight, was detected in both cultivars exposed to simultaneous salinity and drought stresses, with a more pronounced negative effect recorded for the salinity-susceptible rice cultivar (Wei *et al*., 2023). Deciphering the physiological and molecular bases of multiple stress tolerance in rice is crucial for directing future breeding activities (Li *et al*., 2024). This priority becomes an even more difficult task when the targets are seed quality and the existing gap of knowledge concerns the impact of combined stressors on seed physiology.

The impact of multiple stress combinations has been investigated in wheat (*Triticum* spp.), an essential component of the human diet and global food security. Wheat varieties grown under high temperature and CO_2_, as well as under drought and elevated O_3_ levels, revealed deleterious effects on grain yield (Asif *et al*., 2019; Ghosh *et al*., 2020). A multi-factorial experimental system was tested on three European wheat spring varieties, under controlled conditions in a growth chamber, by Galani *et al*. (2022). Combinations of different CO_2_, temperature, and O_3_ levels, delivered in episodic or chronic mode, were designed based on the future climate model projections generated by the Scenario Model Intercomparison Project (ScenarioMIP) and the Coupled Model Intercomparison Project phase 6 (CMIP6) (Tebaldi *et al*., 2021). The effects of these combinations on yield, protein, and dietary mineral nutrient content were analysed, highlighting an increase in grain mineral content (N, Fe, Mg, Mn, P, and Zn) and protein level. The chronic exposure to O_3_ was able to counteract the effects of CO_2_, heat, and drought, possibly due to the role of micronutrients in O_3_-induced physiological responses. Interestingly, plants exposed to O_3_ showed increased N uptake with a constraint in biomass accumulation that resulted in enhanced grain protein content (Wang and Fray, 2011). The study by Galani *et al*. (2022) showed that the combined action of different abiotic stresses associated with climate change negatively impact wheat yield; however, the observed increased accumulation of proteins and most micronutrients may contribute to mitigate yield losses. Notably, the European landrace variety Lantvete showed a remarkable enhancement in nutrients, regarded as an indicator of grain yield plasticity in response to climate stressors, compared with the other tested commercial cultivars (Galani *et al*., 2022). The significant contribution of wheat agrobiodiversity in response to climate stressors, such as O_3_ and heat, reinforces the idea that climate-resilient and high nutritional wheat varieties can be developed through tailored breeding programmes. Breeders’ efforts towards climate-resilient wheat cultivars can be facilitated by mapping the key genome sites and improving marker- and genomic-assisted breeding (Sandhu *et al*., 2022). The meta-analysis performed in wheat by Tanin *et al*. (2022) using quantitative trait loci (QTLs) known to be associated with drought, heat stress, salinity, water-logging stress, pre-harvest sprouting, and aluminum stress allowed the prediction of a total of 134 meta-QTLs for multiple tolerance to five or all six abiotic stresses. Transcriptomics highlighted that 189 genes underlying such meta-QTLs were differentially expressed in response to multiple stress combinations whereas ortho-meta-QTLs among wheat, maize, and rice genomes were identified (Tanin *et al*., 2022). Multi-omics atlases, including physiological, metabolic, hormonal, and transcriptomic data, represent valuable tools for the validation of candidate genes involved in combinatorial stress responses (Da Ros *et al*., 2023). In order to target the key traits for multiple stress tolerance more efficiently, novel multivariate techniques are being developed as reported by Al-Ashkar *et al*. (2023). According to the multi-trait stability index (MTSI), six tolerance (drought, heat stress) multi-indices were calculated for 20 wheat genotypes during three cropping seasons, allowing indentification of those genotypes able to ensure optimal productivity under multiple abiotic stresses (Al-Ashkar *et al*., 2023). Hopefully, knowledge gained from these advanced investigations will contribute to better understanding of those functions directly correlated to seed quality traits.

### Legume grain quality is affected under multiple stress conditions: main players

Concurrent heat and drought stress represents a challenge for cool-season grain legumes, crucial players in sustainable agriculture due to their ability to fix atmospheric nitrogen and their high nutritional value, with severe effects further exacerbated by climate change (Priya *et al*., 2025). Combined heat and drought stress limits grain-filling duration and size, with the strongest impact resulting from drought (Sehgal *et al*., 2019). Lentil (*Lens culinaris* Medik) yields decreased by 43% and 49% under heat stress and combined stresses, respectively (El Haddad *et al*., 2022), with concomitant heat and drought stresses being particularly harmful at the reproductive stage. The recorded seed growth rates decreased by 44–60.2%, whereas seed numbers per plant and seed weights decreased by 35–48.7% and 47–59%, respectively (Sehgal *et al*., 2017, 2019). The nutrient content of lentil simultaneously exposed to both stresses featured low Fe, Zn, and crude protein levels compared with heat stress alone (Choukri *et al*., 2020). On the other hand, tolerant lentil genotypes revealed enhanced antioxidant enzyme activities, polyphenol content, and soluble and reducing sugars levels compared with genotypes sensitive to combined stresses (El Haddad *et al*., 2023). Grain yield was decreased in grasspea (*Lathyrus sativus* L.) exposed to combined drought and heat stress, whereas both stresses led to increased L-oxalyl-2,3-diaminopropionic acid levels and decreasing crude protein content in seeds (Aloui *et al*., 2023). Similar responses were reported in faba bean (*Vicia faba* L.) (Belal *et al*., 2019), common bean (*Phaseolus vulgaris* L.) (Losa *et al*., 2022), and chickpea (*Cicer arietinum* L.) (Awasthi *et al*., 2017). There is limited information concerning the interactions between salinity and temperature and the potential adaptation of seed germination in legume crops. *Medicago sativa* L. germination decreased under high salinity and high temperature conditions, with high temperature being the main inhibitory effect (Sharavdorj *et al*., 2021).

Seed-related phenological traits can support selection for multi-stress-tolerant genotypes, as in the case of lentil QTLs qHt_ss and qHt_ps, linked to seedling survival and pod set (Kumar *et al*., 2018), and chickpea, where meta-QTL-based approaches allowed the identification of *CaLG01* and *CaLG06* loci associated with seed yield performance under stress (Kumar *et al*., 2023). Marker–trait associations (MTAs) for grain grain nutrient content (Fe, Zn, and protein) under stress were described by Samineni *et al*. (2022). Late embryogenesis abundant (LEA) proteins and heat shock proteins (HSPs) were synergically accumulated in wheat in response to combined drought and heat stress, together with stress chaperones controlling protein integrity (Sato *et al*., 2024). Candidate genes associated with combined heat and drought stress in pea (*Pisum sativum* L.) are involved in biotin biosynthesis, actin polymerization, and protein autophosphorylation (Tafesse *et al*., 2020). The state of the art is expanding, however, knowledge on the molecular pathways involved in the response to combined heat and drought stresses is still scanty, due to the limited data available currently, such as those concerning transcriptomics applied to cool-season legume plants exposed to both stresses (Zhang *et al*., 2015).

## Widening the screening for germplasm showing multiple stress tolerance at the level of seed germination

### Focus on minor crops

The screening for multi-stress-tolerant germplasm and its performance at the germination level should be accelerated and assessed for all the agronomically relevant species. A few examples are currently available; in the case of tomato (*Solanum lycopersicum* L.), the screening of both natural accessions and ethyl methanesulfonate-mutagenized lines, under combined drought and heat stress treatments, allowed identificatiom of suitable candidates for developing multi-stress tolerance (Fonseca *et al*., 2024).

Orphan crops or underutilized, neglected species (cereals, legumes, fruit, and root crops), represent ideal targets to explore the response to multiple stresses, such as drought, heat, and salinity in the context of seed quality issues, due to their innate abiotic stress tolerance. They cover an impressive range of agro-biodiversity. Although their pivotal role in ensuring food security and the livelihood of farmers in the most climate-vulnerable regions of the planet has been acknowledged, their relevance in the transition towards sustainable, climate-ready agroecosystems has been pointed out only recently (Verbeecke *et al*., 2023; Akpojotor *et al*., 2025). Several drawbacks are delaying the widespread use of orphan crops, among which is the lack of consistent and advanced scientific knowledge in terms of genetics and physiology, necessary to better characterize those stress tolerance and nutritional traits responsible for improved quantity and quality yields. In most cases, low yield and limited marker expansion are the main cause of the poor levels of adoption in major farming systems (Verbeecke *et al*., 2023). To date, seed quality issues in orphan crops have been discussed to highlight the need for tailored guidelines in terms of seed security, in agreement with the Food and Agricultural Association (FAO) framework and parameters (seed availability, seed access, varietal suitability, and seed quality) (Mwangi *et al*., 2020).

### Tailored seed priming applications in minor crops for multiple stress tolerance: promising hints

Seed priming has been suggested as a reliable strategy that can contribute to overcome the scanty seed production observed in neglected species; however, this ambitious goal will require the synergic integration of knowledge in seed biology, technology, and agronomy, and a strong focus on the potential of this technique in promoting the response to multiple stresses. In this context, protocols available for different major crops will guide researchers towards tailored applications in orphan crops (Balestrazzi *et al*., 2024). Only a few reports are currently available in orphan crops describing the seed response under abiotic stress, such as in the case of grass pea (Tokarz *et al*., 2020; Wiraguna *et al*., 2020), millet [*Eleusine coracana* (L.) Gaertn.] (Divya *et al*., 2022), and fenugreek (*Trigonella foenum graecum* L.; El-Rafasi *et al*., 2021; Hussain *et al*., 2022); however, to our knowledge, research on multiple stressors is still poorly represented. On the other hand, information about the potential of different seed priming agents applied to orphan legumes as tools to enhance tolerance to single stresses is expanding. Hojjat (2020) demonstrated the efficacy of nanopriming as a strategy to enhance salt stress tolerance in grass pea. Titanium dioxide nanoparticles were able to boost germination and seedling growth under salt stress, promoting accumulation of the osmoprotectant proline. Goufa *et al*. (2025) assessed the beneficial effects of different seed priming treatments (hydropriming, biopriming, and hybrid priming, resulting from the combination of both treatments) on the agronomic performance of two grass pea varieties under salt stress (80 mM and 160 mM NaCl). Biopriming significantly improved the root and shoot growth performance of both varieties under salt stress. Pagano *et al*. (2025) explored the potential of hydropriming as a tool to mitigate the heat stress-mediated impact on the germination performance of different grass pea, *Pisum sativum* var*. arvense* (forage pea), and fenugreek accessions. The accession-specific beneficial impact of hydropriming under heat stress conditions was revealed. In grass pea seeds, the alkaline comet assay revealed DNA damage accumulation in response to heat stress, and the repair dynamics promoted by hydropriming. DNA repair and antioxidant genes were positively modulated in hydroprimed seeds under heat stress. Successful seed priming protocols have been reported for millets. Silicon-based seed priming was applied to enhance salt stress tolerance in two finger millet (*E. coracana*) landraces by boosting antioxidant enzyme activities and preventing lipid membrane damage (Shaikh *et al*., 2024). Silicon supplementation increased the chlorophyll content, and promoted the exclusion of Na^+^ while maintaining a proper concentration of K^+^ and Ca^2+^ under salinity conditions. Halopriming carried out with NaCl solutions could improve the germination performance of finger millet, little millet (*Panicum sumatrense* Roth), and barnyard millet (*Echinochloa esculenta* L.) challenged with salt stress (Venothini *et al*., 2024). The treatment was effective at the level of seedling establishment, resulting in an overall enhanced stress tolerance. A range of seed priming treatments based on the use of mannitol, KCl, KNO_3_, CaCl_2_, *Parthenium*, neem (*Azadirachta indica* L.), and tulasi (*Ocimum tenuiflorum* L.) leaf extracts were successfully tested by Arun Kumar Chaurasia *et al*. (2021). The efficacy of the treatments was assessed with field trials, and the agronomic performance was evaluated at the growth and reproductive stages, particularly in terms of number of fingers per panicle and seed yield. The simultaneous exposure to drought and salinity stress was tested on transgenic finger millet plants expressing the bacterial *mtlD* gene encoding mannitol-1-phosphate dehydrogenase within the mannitol biosynthetic pathway (Hema *et al*., 2014). The transgenic lines proved tolerant to the combined stresses, highlighting the key role of mannitol as a protective osmolyte. The effects of combined polyethylene glycol (PEG)-induced water stress and high temperature were investigated by Kastury *et al*. (2024) in 30 finger millet genotypes. The study identified the best performing genotypes under the combined stress conditions, providing valuable germplasm for developing enhanced tolerance towards multiple abiotic stresses. Kannababu *et al*. (2025) analysed the response to accelerated ageing and seed longevity traits across 221 different finger millet accessions, using a genome-wide association approach (GWAS). The resulting MTAs for 27 traits and functional annotation revealed the role of specific genes that influence seed coat integrity, seed mechanical sensing, and stress adaptation response. Such findings represent the starting point for more focused strategies based on stress tolerance and quality seed traits that can be eventually translated to other neglected species (Kannababu *et al*., 2025). The screening for genotypes with enhanced tolerance to a range of abiotic stresses will reveal potential candidates harbouring multiple stress tolerance that protect plants during their entire life cycle, including early developmental stages and germination. Once the most tolerant germplasm has been identified, pre-sowing techniques can further boost the endogenous abiotic stress tolerance.

### Advanced tools are required to explore MFSC: high-throughput phenotyping and omics

**Fig. 1.**
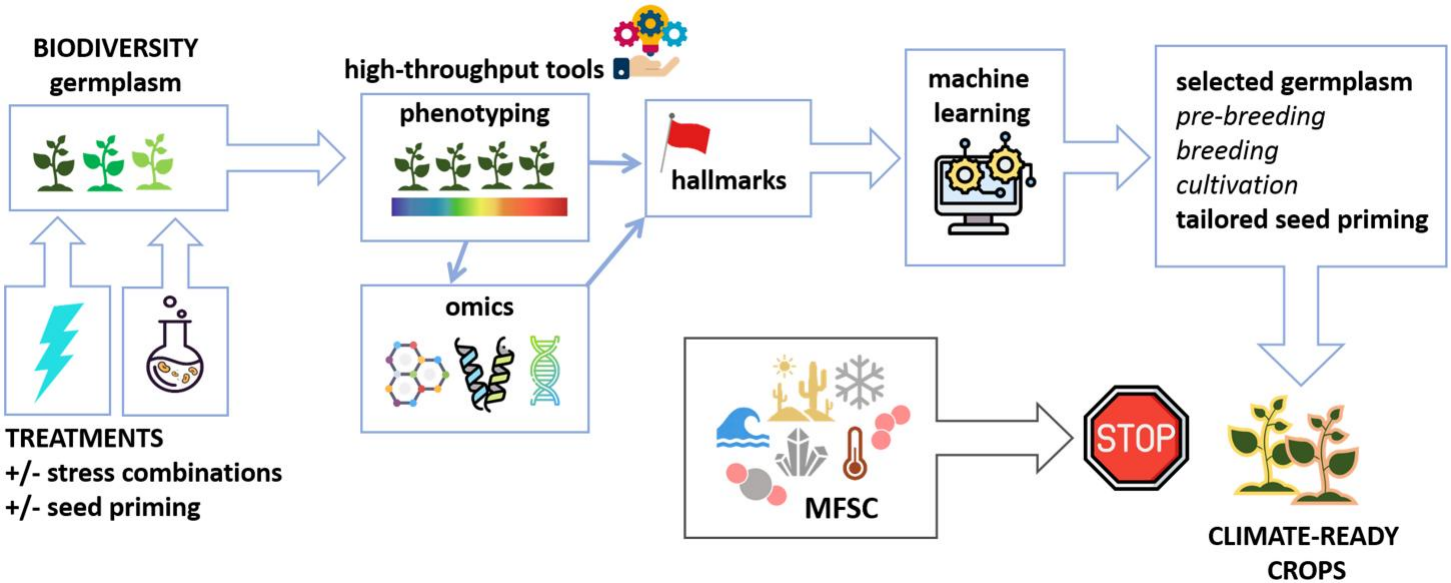
Schematic representation of the roadmap leading to the development of climate-ready crops with multiple stress tolerance. Germplasm collections represent valuable and unique sources of biodiversity in terms of abiotic stress tolerance. Working systems designed to assess the degree of stress tolerance/sensitivity as well as for testing the current seed priming protocols will undergo high-throughput analysis to select the best performing genotypes/accessions. Phenotyping at the seed, seedling, and plant level is required to select the materials for omics-based investigations. This will lead to the identification of candidate seed quality hallmarks. Results from this multi-level analysis will be integrated into dedicated databases and used to feed algorithms for machine learning approaches.

The development of novel varieties tolerant to combined abiotic stresses is progressing at limited speed, and only the use of advanced tools will produce a significant acceleration of the work of the breeders. Phenotyping, a critical step for the identification of potential donors of resilience traits, is now expanding at the seed level, with an increasing range of protocols now available for delivering combined stresses during germination and to target the specific growth stages that are most stress sensitive (Fig. 1). Kota *et al*. (2023) developed a standardized screening method to assess the response of rice cultivars to combined drought and salinity stresses and in parallel to each separate stress. Different timing of drought and salinity application as well as increasing stress intensities were applied, resulting in an optimized protocol in which seeds were sown in saline soil at 75% of field capacity and then exposed to progressive dry conditions (Kota *et al*., 2023). Phenotyping at the seed level is based on the analysis of seed weight, size, and number, a demanding procedure when high numbers of different genotypes must be screened. Schmidt *et al*. (2020) employed computed tomography to monitor changes in wheat seed features, such as seed shrivelling and germ deformation, as indicators of the wheat response to combined drought and frost stress, developing a screening protocol compatible with the large-scale analysis required to support breeding programmes. Multi-omics and systems biology, integrating genomics, transcriptomics, proteomics, metabolomics, and epigenomics, provide a unique perspective to effectively study these extremely complex mechanisms with high-resolution tools (Fig. 1). Multi-omics integration can be used to link phenotype to genotype and build predictive models for precision breeding, and this perspective, currently applied to investigate the plant response to single abiotic stresses, could be translated to decipher the MFSC issues (Roychowdhury *et al*., 2023). A few reports show the efficacy of such an approach as an informative tool to describe seed pre-germinative metabolism and particularly the DDR dynamics reflecting the seed repair response or the deleterious effects of genotoxic damage (Araújo *et al*., 2019; Pagano *et al*., 2019).

In the attempt to provide a wider picture of the molecular networks underlying the multiple stress response at the germination stage, omics profiles might be used as a source of preliminary information, as a proxy of the potential for multiple stress tolerance held by specific genotypes.

The seed transcriptome of the highly stress-tolerant species *Solanum paniculatum* L. has been investigated in order to reveal the molecular changes associated with osmopriming and the beneficial effects of this treatment in terms of enhanced drought tolerance (da Silva *et al*., 2023).

According to this study, 10% of the differentially expressed genes encoded proteins involved in the abiotic stress response, mainly water, oxidative, saline, and heat stresses. Such a picture becomes a valuable background for the identification of molecular hallmarks useful to support the screening for germplasm with high-quality seeds and stress-tolerant germination performance. Furthermore, players showing concomitant up-regulation may be part of a potential cross-tolerance mechanism or contribute to increased multiple stress tolerance (da Silva *et al*., 2023). PEG-mediated osmopriming provided protection against multiple abiotic stresses as reported for asparagus (*Asparagus officinalis* L.) and spinach (*Spinacia oleracea* L.) seeds exposed to combined drought and low temperature stresses (Bittencourt *et al*., 2004; Chen *et al*., 2010), as well as sunflower (*Helianthus annuus* L.) seeds challenged with drought and salinity stress (Moghanibashi *et al*., 2015). The knowledge gathered on the performance and potential of seed priming to promote multiple stress tolerance should be integrated with the valuable information provided by the study of cross-stress tolerance mechanisms.

## Seed priming promotes multi-stress tolerance

### The potential of seed priming in the response to MFSC

In the most climate-vulnerable regions of the planet, crop plants are easy targets of multiple environmental stresses. The need for short-term approaches able to improve crop performance under the severe effects of climate stressors has led to an increased focus on those pre-sowing techniques, known as seed priming, able to enhance seed vigour and seedling stress tolerance, promoting successful crop stand establishment under challenging conditions. Seed priming is considered as a simple and inexpensive tool to maximize seed performance in a wide range of targets, both agronomically relevant crops and endangered native species (Devika *et al*., 2021; Pagano *et al*., 2023; Hernández *et al*., 2024) (Box 1).

### Seed priming agents are versatile tools for targeted applications

Water is the simplest priming agent, however, a wide range of chemicals, bioactive compounds, and microorganisms are currently used to develop formulations for tailored seed priming protocols (Pagano *et al*., 2023) (Box 1). Hydropriming represents the most cost-effective and eco-friendly priming protocol, exclusively based on the use of water, with demonstrated capability to boost stress tolerance (Tanwar *et al*., 2023; Zhang *et al*., 2023). Osmopriming, also known as halopriming, is based on the use of aerated solutions containing salts or PEG at different water potentials, resulting in mild water stress conditions that trigger the seed repair response and promote osmoprotective mechanisms. The efficacy of osmopriming agents for improving seed germination and seedling establishment under adverse environmental conditions has been documented (Lei *et al*., 2021; Ma *et al*., 2024). Chemopriming encompasses a range of micronutrients, organic molecules, and primary or secondary natural metabolites, such as mannose and mannitol (Hameed and Iqbal, 2014), silicon (Hameed *et al*., 2021), zinc, and boron (Ebrahim Pour Mokhtari *et al*., 2022), whereas in the case of redox priming, specific compounds involved in redox mechanisms are used, such as hydrogen peroxide (H_2_O_2_), the NO donor sodium nitroprusside, and spermidine (Fuchs *et al*., 2023; Iqbal and Yaning, 2024). Hormopriming relies on phytohormones as priming agents, and on their role as chemical messengers in signal transduction pathways and crosstalk. Phytohormones can mediate the stress response as well as growth and development in germinating seeds, contributing to mitigation of abiotic stresses (Kaler *et al*., 2024). Biopriming is applied using plant growth-promoting bacteria able to promote germination and provide additional benefits on seedling growth (Fiodor *et al*., 2023). Biopriming is also performed using beneficial fungi able to enhance plant growth and to increase nutrient uptake and stress tolerance (Aizaz *et al*., 2023). Nanopriming represents a relevant nanotechnology application in agroecosystems, able to trigger stress tolerance responses (Lee and Kasote, 2024). Nanoparticles are delivered and deposited on the seed surface where they can either adhere or enter and distribute within the seed tissues, exerting their beneficial action, such as enhanced water uptake, antioxidant machinery modulation, and reserve mobilization (Shelar *et al*., 2024). Green-synthesized nanoparticles, based on the use of phytochemicals, have been developed as an environmentally friendly approach that avoids the need for toxic reagents (Aggarwal *et al*., 2025). Physical methods for seed priming represent another area of interest in seed technology. These eco-friendly and cost-effective treatments can be performed using ionizing radiation (e.g. X-rays and γ-rays) and non-ionizing radiation (e.g. ultrasonic waves, magnetic fields, microwaves, and infrared light) (Bera *et al*., 2022). Priming with UV radiation proved effective, not only enhancing the germination rate and biomass production, but also triggering improved photosyntesis and the antioxidant response under stress conditions (Escobar-Hernández *et al*., 2024).

### Hybrid priming as a synergistic upgrade of priming technology

Combination of individual seed priming protocols (hybrid priming) is regarded as an effective strategy to synergically increase seed vigour under adverse conditions (Box 1). Hybrid priming with a laser and H_2_O_2_ increased salt tolerance and improved the antioxidant value of *Salvia officinalis* L. plants (Amooaghaie *et al*., 2024) whereas hydro-electro hybrid priming was able to promote the germination of carrot (*Daucus carota* L.) seeds (Zhao *et al*., 2021). The potential of hybrid priming as a booster of multiple stress tolerance still needs to be assessed. This will be a difficult task, considering the multi-layer composition of hybrid priming versus concomitant multiple stressors. The current state of the art shows that individual seed priming treatments can provide protection against the simultaneous exposure to different abiotic stresses. Fu *et al*. (2024) assessed the beneficial impact of melatonin-based seed priming on wheat seedling growth and, in particular, root physiological performance, under combined drought and salinity stress delivered through PEG- and NaCl-based treatments. Melatonin-mediated priming enhanced root hydraulic conductivity under combined PEG and NaCl treatments, strengthening multi-stress tolerance at the level of the root system. Zhu *et al*. (2021) reported the results of hormopriming and chemopriming applied to rapeseed (*Brassica napus* L.) varieties exposed to cold and drought stress, using phytohormones, namely salicylic acid, gibberellic acid, and ABA, as well as chemopriming with sodium nitroprusside and calcium chloride. All the tested treatments significantly improved the germination performance and seedling growth, promoting superoxide dismutase and ascorbic peroxidase levels under stress conditions (Zhu *et al*., 2021). Despite these promising results, the number of reports that show the efficacy of seed priming against multiple stress combinations is still too limited and, in most cases no information was provided concerning the molecular targets and processes involved (Table 1).

**Table 1.** List of seed priming treatments that proved effective in providing enhanced tolerance to combined stresses

Seed priming (priming agent)	Target species	MFSC	Target/effect	References
Hydropriming	*Helianthus annuus* L.	Drought, salinity	Free proline	Moghanibashi *et al*. (2015)
Osmopriming				
(PEG6000, sea water)	*Asparagus officinalis* L.	Drought, heat		Bittencourt *et al*. (2004)
(PEG8000)	*Spinacia oleracea* L.	Drought, heat		Chen *et al*. (2010)
(PEG6000)	*Helianthus annuus* L.	Drought, salinity	Free proline	Moghanibashi *et al*. (2015)
Chemical priming				
(melatonin) (SNP) (CaCl_2_)	*Triticum aestivum* L. *Brassica napus* L.	Drought, salinity	Lipid peroxidation, proline, soluble protein/sugar, SOD/POD/CAT activity, aquaporin gene expression, SOD/APX/POD/CAT activity	Fu *et al*. (2024) Zhu *et al*. (2021)
Hormopriming				
(SA) (GA) (ABA)	*Brassica napus* L.	drought, cold	SOD/APX/POD/CAT activity	Zhu *et al*. (2021)

MFSC, multi-factorial stress combination; PEG, polyethylene glycol; SOD, superoxide dismutase; POD, peroxidase; APX, ascorbate peroxidase; CAT, catalase; SNP, sodium nitroprusside; SA, salicylic acid; GA, gibberellic acid; ABA, abscisic acid.

## Building the protective response to multi-factorial stress combination through cross-stress tolerance mechanisms

Tolerance to multiple stresses can be achieved by triggering cross-stress tolerance or cross-protection (Liu *et al*., 2022; Rodgers and Gomez Isaza, 2023). In this case, the pre-exposure to a primary stress improves the plant growth performance when a secondary stress is subsequently applied. Such adaptive changes are also defined as *cis*- or *trans*-priming, depending on whether the secondary stress factor is the same or a different one. In this context, Liu *et al*. (2022) refer to seed priming as a primary stress, able to establish a cross-stress memory. Cross-protection can be considered as a sort of pre-adaptation to global change stressors; however, the costs in terms of fitness still need to be quantified. Indeed, a ‘cross-protective’ phenotype associated with excessive metabolic energy consumption may not represent a sustainable option, and this can be verified by assessing interactions with multiple stressors at the field level under changing environments as well as under controlled conditions (Rodgers and Gomez Isaza, 2023).

The successful output of this approach relies on the proper synergistic interaction between pathways and mechanisms at the forefront of the stress adaptation response, such as antioxidant and glyoxalase systems (Hossain *et al*., 2018). Up-regulation of fundamental antioxidant enzymes, including ascorbate peroxidase, superoxide dismutase, catalase, monodehydroascorbate reductase, dehydroascorbate reductase, and glutathione reductase, is part of the seed repair response (Bailly, 2019). The glyoxalase system includes glyoxalase I and glyoxalase II enzymes that convert the toxic methylglyoxal into the non-toxic product D-lactate. Subsequently, lactate dehydrogenase catalyses the conversion of D-lactate into pyruvic acid that enters the tricarboxylic acid cycle (Li, 2016). Endogenous methylgyoxal levels increase in plants under abiotic stress conditions, triggering tolerance mechanisms through crosstalk with ROS, Ca^2+^, and ABA (Li, 2016).

Synergistic co-activation of the glyoxalase and antioxidant systems is a key feature of cross-stress tolerance in plants (Li, 2016). Both systems depend on common components such as glutathione. The interdependence of the glyoxalase pathway and glutathione metabolism has been demonstrated. The modulation of the glyoxalase systems is concomitant with enhanced accumulation of non-enzymatic antioxidants, particularly ascorbate (Sezgin Muslu and Kadioglu, 2021) and phenolic compounds (Talaat and Todorova, 2022). The impact of different priming agents, such as salicylic acid (hormopriming), ascorbic acid (chemical priming), and NaCl (osmopriming) on the ability of wheat seeds to germinate under drought stress was reported by Alam *et al*. (2022). Primed seeds showed enhanced germination performance under stress, associated with decreased ROS accumulation and increased antioxidant response, as well as enhanced activity of glyoxalase I and glyoxalase II (Alam *et al*., 2022). Similarly, osmopriming (CaCl_2_) and hormopriming (salicylic acid) applied to canola seeds enhanced the glyoxalase system and antioxidant response, thus improving ROS and methylglyoxal detoxification (Ashraf *et al*., 2022). The exogenous application of methylglyoxal to wheat seed was able to improve germination seedling performance under salt and Cd stresses, respectively, by triggering the antioxidant and glyoxalase defense response and modulating osmolyte biosynthesis (Li *et al*., 2019).

Small HSPs (sHSPs), required to maintain cellular homeostasis and prevent the accumulation of misfolded proteins and aberrant aggregates, also contribute to cross-stress tolerance. The overexpression of sHSPs is associated with enhanced antioxidant activities, osmolyte levels, and expression of stress-responsive genes, indicative of cross-stress tolerance mechanisms (Do *et al*., 2023). sHSPs contribute to protect seeds against desiccation stress, preventing aberrant protein aggregation and assisting in the refolding of denatured proteins during imbibition (Kaur *et al*., 2015).

The knowledge landscape shaped by researchers’ efforts addressing seed quality issues in relation to multiple climatic stressors, covering molecular mechanisms involved in MFSC response, could be significantly enriched by looking at the way in which seeds cope with genotoxic damage resulting from combined stress exposure. DDR represents a key player in this context, since oxidative damage generated by the uncontrolled ROS enhancement under a wide range of abiotic stresses is converted into genotoxic damage at the nuclear level, posing a risk to the integrity of genetic information.

## The DNA damage response machinery

### Overview

The DDR molecular machinery is structured as a multi-level cascade of events mediated by sensors, transducers, mediators, and effectors that undergo a tight spatio-temporal regulation (Szurman-Zubrzycka *et al*., 2023). Sensing is performed by the MRN (MRE11, meiotic recombination 11; RAD50, radiation sensitive 50; NBS1, Nijmegen breakage syndrome 1) complex that binds DSBs (double-strand breaks), and RPA (replication protein A) that targets SSBs (single-strand breaks) (Dueva and Iliakis, 2020). Upon DNA damage sensing, the highly conserved protein kinases ATM (ataxia telangiectasia mutated) and ATR (ATM and RAD3-related) trigger the downstream responses mediated by the master regulator SOG1 (suppressor of gamma 1), the plant homologue of the mammalian tumour suppressor p53 (Sakamoto *et al*., 2021), whereas other SOG1-independent pathways have been characterized, acting in parallel or through complex crosstalk along the DDR cascade (Casati, 2023). The ATM kinase is recruited by the MRN complex at the DSB sites where it promotes H2AX histone phosphorylation and the consequent engagement of the DNA repair machinery, whereas ATR kinase is responsive to SSBs and stalled replication forks. ATM can also be activated by oxidative stressors, such as H_2_O_2_, through MRN-independent pathways (Zhao *et al*., 2024). Based on the severity of genotoxic lesions, seeds face two options, either blocking the cell cycle and engaging the multiple DNA repair pathway that specifically target the different types of lesions, or triggering programmed cell death (PCD). The latter will allow the selective removal of cells carrying high DNA damage, that will compromise the development of healthy seedlings (Szurman-Zubrzycka *et al*., 2023).

The involvement of DNA repair pathways in the plant response to abiotic stress has been documented, with a recurrent focus on specific players (Cimini *et al*., 2019; Szurman-Zubrzycka *et al*., 2023). Those DDR components involved in the response to single climatic stressors (e.g. drought, heat, and salinity) can be envisaged as potential candidates acting within a more complex response to multiple stresses (Dorn and Puchta, 2020). Such a view can be translated to seeds since there are reports dealing with the specific contribution of these DDR players to seed quality (Sincinelli *et al*., 2025).

### DDR relevance in seed germination

Genome maintenance in seeds is fundamental for preserving germplasm stability in the long term, ensuring the safe replication of genetic material. The DDR is a crucial component of seed pre-germinative metabolism, necessary to mitigate the impact of oxidative lesions on genome integrity, by removing deleterious mutations accumulated during the seed life cycle, from development and maturation, during storage, until imbibition starts with the resumption of metabolism (Waterworth *et al*., 2019, 2022, 2024; Pagano *et al*., 2023; Sincinelli *et al*., 2025). In the desiccated state, seeds preserve cellular homeostasis by retaining minimal metabolic activities, but cellular structures and macromolecules are subjected to progressive injury (El-Maarouf-Bouteau, 2022). Deteriorated seeds show delayed germination; the process is extended through a lag phase whose duration reflects the time required to properly repair DNA lesions (Waterworth *et al*., 2019). DNA repair pathways are switched off at the end of germination, when radicle protrusion occurs and the genetic programme for seedling development is activated. The loss of DNA repair activity is concomitant with the resumption of cell cycle progression in embryo cells (Waterworth *et al*., 2022). The link between DDR and seed physiology paves the way to mitigation strategies against environmental stresses affecting crucial seed quality components, such as elevated germination performance and extended longevity. DDR can be envisaged as a source of possible markers for crop improvement through breeding, biotechnology, and seed vigorization protocols (Pagano *et al*., 2017, 2019, 2023; Sincinelli *et al*., 2025). The contribution of specific DDR players to the response against a single abiotic stress has been documented for drought (Shim *et al*., 2018; Jaiswal *et al*., 2021; Poku *et al*., 2021; Sihi *et al*., 2022; Mohapatra *et al*., 2023), salinity (Sihi *et al*., 2022; Tang *et al*., 2022; Mohapatra *et al*., 2023; Wang *et al*., 2023; Mahapatra and Roy, 2024; Chirinos-Arias and Spampinato, 2025), and temperature stress (Han *et al*., 2021; Pagano *et al*., 2025). Evidence on the direct implication of DDR components in the stress response is also provided by the extensive research carried out using DDR mutants (Roy and Das, 2017; Vladejić *et al*., 2024; Kim *et al*., 2025; Szurman-Zubrzycka *et al*., 2025). This growing scientific background should aid the efforts towards understanding whether and how DDR players may converge to promote a synergic action to better protect genome integrity under the harmful effects of combined climatic stressors.

### Effective DDR is a proxy of seed quality

A deeper understanding of the role played by DDR components in the dynamics of pre-germinative metabolism will promote a better understanding of multi-stress tolerance in seed germination. Damaged bases are processed by the BER (base excision repair) pathway, where different DNA glycosylases targeting a range of specific lesions generate an abasic site. The latter is subsequently removed and the resulting gap is filled via DNA synthesis (Roldán-Arjona *et al*., 2019; Grin *et al*., 2023). One of the major products of DNA oxidation, the oxidized nucleotide 8-oxoguanine (8-oxoG), is removed by either 8-oxoguanine DNA glycosylase/lyase (OGG1) or formamidopyrimidine-DNA glycosylase (FPG) (Roldán-Arjona *et al*., 2019; Grin *et al*., 2023). Increased 8-oxo-dG levels were detected during seed imbibition in *Medicago truncatula* Gaertn. seeds (Macovei *et al*., 2010) and, accordingly, both *MtOGG1* and *MtFPG* genes were significantly up-regulated (Macovei *et al*., 2011). The number of oxidative lesions was further enhanced when seeds were imbibed under osmotic stress conditions (Balestrazzi *et al*., 2011). Arabidopsis transgenic lines overexpressing the *AtOGG1* gene showed enhanced tolerance to seed ageing, and higher seed viability when germination was carried out at elevated temperatures or in the presence of salt stress, compared with control lines (Chen *et al*., 2012). Soybean (*Glycine max* L.) seed lots with divergent vigour profiles revealed expression patterns of the *GmOGG1* gene that positively correlated with germination performance (Ducatti *et al*., 2022). The expression profiles of *OGG1* and *FPG* genes were assessed in spring varieties of barley (*Hordeum vulgare* L.), wheat, and rye (*Secale cereale* L.) during seed germination. Species-dependent patterns were observed, with gene transcripts peaking at different time points, possibly reflecting differences in seed coat structure and water uptake kinetics (Kowalik and Groszyk, 2023). Based on these findings, OGG1 and FPG may represent ideal candidates with a potential role in DDR under combined abiotic stresses. Bulky DNA damage, namely those helix-distorting lesions that impair replication and transcription, are removed by the NER (nucleotide excision repair) pathway, based on the excision of the aberrant strand, and the subsequent gap filling, mediated by DNA polymerases (Cohen and Adar, 2023; Szurman-Zubrzycka *et al*., 2023). Among the players in NER implicated in seed germination, there is a member of the xeroderma pigmentosum complementation group proteins (XPs) required to coordinate the GG (global genome)–NER and TC (transcription-coupled)–NER subpathways. The Arabidopsis *atxpb1* plant line carrying a mutation in the *AtXPB1* gene showed a low seed germination rate and absence of germination synchrony, as well as delayed organ development (Costa *et al*., 2001). Highly cytotoxic DSBs, occurring during DNA replication and under oxidative stress, are repaired by non-homologous end joining (NHEJ) or homologous recombination (HR) (Charbonnel *et al*., 2011; Vitor *et al*., 2020). NHEJ comprises classical NHEJ (cNHEJ) and alternative NHEJ (aNHEJ; also known as microhomology-mediated end-joining). The damaged ends, detected by repair factors, are joined through the KU70/KU80 heterodimer that allows enzyme-mediated end-processing reactions until the final ligation takes place. NHEJ players have been connected with seed quality, as in the case of Arabidopsis mutants with defective *AtLIG4* and *AtLIG6* genes, involved in cNHEJ and aNHEJ, respectively. The *lig4* and *lig6* mutant lines produced seeds hypersensitive to accelerated ageing, thus highlighting the link between NHEJ and seed longevity, and the specific role of both subpathways in preserving genome integrity in seeds (Waterworth *et al*., 2010). In Arabidopsis KU70-deficient lines, seeds proved hypersensitive to the alkylating agent methylmethane sulfonate (MMS), and this finding provided evidence for the requirement for NHEJ specifically at the imbibition stage in response to DNA damage accumulation (Riha *et al*., 2002). HR is based on homology search and DNA strand invasion mediated by the Rad51–ssDNA pre-synaptic filament (Wright *et al*., 2018). Mutant maize lines lacking a functional RAD51 protein showed delayed germination and high seedling mortality, compared with wild-type lines (Li *et al*., 2008). The role of the DNA recombinase DMC1 (DNA meiotic recombinase 1), involved in DSB repair, has been investigated in the context of seed germination using *B. rapa* transgenic lines showing RNAi-mediated down-regulation of the *BrDMC1* gene. When seeds were exposed to salt stress, the BrDMC1-RNAi lines showed a significant decrease in germination rate, compared with control lines, indicating the requirement for DMC1 under adverse environments (Wang *et al*., 2023). Genome maintenance is also mediated by repair pathways able to remove DNA–protein cross-links, such as those caused by DNA topoisomerases (Enderle *et al*., 2019; Hacker *et al*., 2022). TDP1 and TDP2 (tyrosyl DNA-phosphodiesterase) enzymes process the irreversible protein tyrosyl-DNA complexes involving topoisomerases I and II, respectively. However, their roles have been expanded since TDP1 is involved in the repair of oxidative damage-induced 3'-phosphoglycolates and alkylation damage-induced DNA breaks, whereas TDP2 is able to resolve topo II–DNA adducts (Pommier *et al*., 2014). The *M. truncatula MtTdp1α* and *MtTdp1β* genes were significantly up-regulated during imbibition (Macovei *et al*., 2010; Balestrazzi *et al*., 2011), whereas subsequent *in silico* studies provided evidence of gene-specific transcriptional responses occurring within Arabidopsis seeds (Pagano *et al*., 2023). Data collected from seeds imbibed in water revealed how *AtTdp1α* and *AtTdp1β* gene transcripts peaked during early and late imbibition, respectively, while phytohormone treatments showed that both *AtTdp1α* and *AtTdp1β* genes were up-regulated by GA and down-regulated by ABA, in agreement with their key roles in seed germination (Pagano *et al*., 2023).

The knowledge so far gained about the essential role of DDR in plants must be expanded through in-depth investigation, and we are still far from having a complete picture of the DDR potential in promoting multiple stress tolerance in seeds. In this unclear scenario, promising hints come from those studies that explore the response of pre-germinative metabolism to seed priming and the impact of treatments on DDR mechanisms.

## Seed priming and DNA damage response provide an eclectic experimental system to investigate the seed response to multi-factorial stress combination

Seed priming represents a valuable source of information on the way in which specific components of the DDR can be boosted, contributing to enhanced germination performance and seedling stress tolerance. Thus, it is possible to build up dedicated profiles of those DDR players most frequently associated with the genotoxic stress response in seeds, further corroborating their contribution under the accelerated metabolic conditions triggered by priming agents. Such profiles can then be expanded if experimental evidence exists about the involvement of components of the DDR in the repair response of primed seed challenged with either separate or combined abiotic stresses. In this context, the current gap in knowledge is quite consistent; however, there are a few DDR players emerging as models that could drive specific research on multiple stress tolerance.

Some of the components of DDR cited in the previous paragraphs, whose beneficial role in the seed repair response has been demonstrated, proved to be effective indicators of successful seed priming. The *OGG1* gene is the most represented in the literature. When hydropriming was applied to eggplant (*Solanum melongena* L.) seeds with a temporal range from 24 h to 96 h, the *SmOGG1* gene showed up-regulation within the first 24 h of controlled imbibition and, subsequently, a peak in *SmOGG1* transcript occurred during dry-back (Forti *et al*., 2020a). The level of *SmOGG1* transcript was in most cases higher in high-quality seed lots, compared with low-quality samples. Up-regulation of the *SmOGG1* gene was also detected in hydroprimed eggplant seeds at 2 h of post-priming imbibition (Forti *et al*., 2020a). In their report, Kiran *et al*. (2020) described the up-regulation of DDR genes in imbibed eggplant seeds primed with NaCl, namely NHEJ and MMR (mismatch repair) genes, such as *SmKU70* and *SmMSH2* (mutS homologue 2), respectively, as well as the *SmOGG1* gene. Up-regulation of *MtOGG1* and *MtFPG* genes was also observed in *M. truncatula* seeds treated with hydropriming and biopriming and then tested using degraded agricultural soil from abandoned areas in India (Forti *et al*., 2020b). The potential of hydropriming to mitigate the impact of heat stress on seed germination in two different grass pea accessions has been investigated in terms of DDR mechanisms by Pagano *et al*. (2025). The DDR genes *LsOGG1*, *LsFPG*, and *LsLig*, encoding DNA glycosylases and a DNA ligase involved in BER, were up-regulated in unprimed seeds exposed to heat waves in both accessions. Up-regulation was also observed in primed seeds challenged with heat waves but only in one accession, thus suggesting some genotype-dependent effect (Pagano *et al*., 2025).

## Role of signalling molecules in DNA damage response, seed priming, and response to multi-factorial stress combination

The molecular networks ruled by the signalling molecules H_2_O_2_, NO, and hydrogen sulfide (H_2_S) represent an intriguing research target in seed biology, as a potential source of candidate hallmarks that could be used to promote the response to MFSC at the germination level. H_2_O_2_, a long-lived ROS that can easily diffuse across membranes, is involved in crucial crosstalk events with plant phytohormones, such as ABA, gibberellins, and ethylene, as well as with NO and H_2_S (Wojtyla *et al*., 2016). To allow such a signalling role, H_2_O_2_ accumulation within the seed tissues is under tight control by the antioxidant machinery to guarantee the proper balance between antioxidant signalling and cytotoxic damage, as described in the ‘oxidative window’ model (Bailly, 2019). Both NO and H_2_S are volatile molecules that contribute to modulate seed germination with similar functions and, depending on their respective level, they can behave in an antagonistic or synergic manner (Corpas *et al*., 2019). NO controls ABA-dependent signalling cascades targeting seed dormancy, germination, and seedling growth (Signorelli and Considine, 2018) whereas endogenous H_2_S is accumulated during the early stages of seed imbibition (Baudouin *et al*., 2016). Cysteine-rich proteins can be regulated through post-translational modification, such as NO-mediated *S*-nitrosation and persulfidation, caused by H_2_S (Corpas *et al*., 2019).

**Fig. 2.**
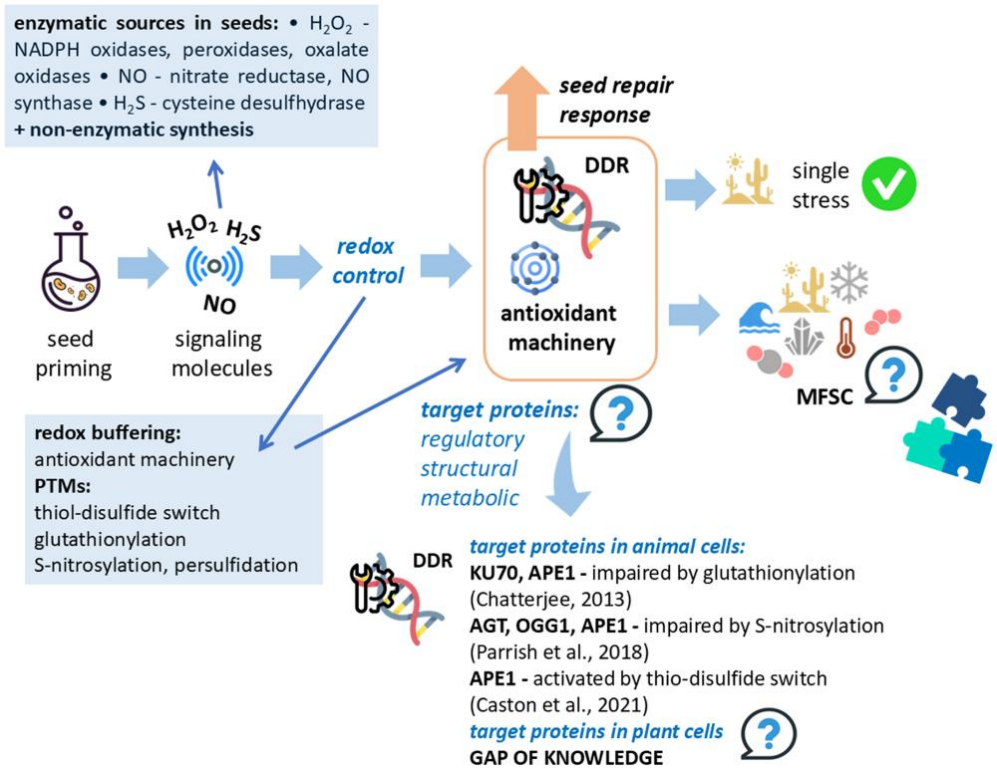
The role of signalling molecules in the context of seed pre-germinative metabolism and, particularly, under priming treatments still needs to be deciphered. A consistent gap in knowledge concerns the redox control exerted by these signalling molecules in the frame of DDR. State-of-the-art information available in animal cells could guide parallel investigations in seeds.

How do these signaling pathways connect to DDR? Studies from animal cells highlight that redox signalling influences DNA repair by inducing redox-based changes in factors involved in DNA repair. Indeed, redox modifications enhance the activity of a key DDR player, the protein kinase, fostering activation of DNA repair pathways (Li *et al*., 2025). Similarly, redox modifications involve other proteins involved in DSB repair, such as Ku and DNA-PKcs (DNA-dependent protein kinase catalytic subunit) (Alnajjar and Sweasy, 2019). In plants, redox-dependent regulatory mechanisms include redox buffering and post-translational modifications, such as the thiol–disulfide switch, glutathionylation, and *S*-nitrosation (Cimini *et al*., 2019), and it is expected that H_2_S and NO prevent DNA damage either through ROS scavenging or by modifying the components of DDR to enhance their activity, as documented in animal systems. The review by Huang *et al*. (2021) provides an overview of the interactions between ROS, NO, and H_2_S in plants, in relation to DNA damage, focusing on the impact on mitochondrial DNA repair. The current state of the art should be translated to model systems such as seed germination and seed priming to assess the role of NO and H_2_S in mitigating genotoxic damage. This will allow expansion of current knowledge on the molecular determinants that link DDR and seed priming, paving the way to novel potential seed quality hallmarks (Chen *et al*., 2019; Sharma *et al*., 2022). Seed priming with signalling molecules has been successfully applied in a wide range of species, enhancing stress tolerance. The beneficial effects of H_2_O_2_ priming applied to seeds have been reported, including its ability to enhance antioxidant defences, to balance ROS production and scavenging, and to promote accumulation of osmoprotectants, as well as the impact of gene expression and signalling (Choudhary *et al*., 2024; Jamshidi Goharrizi *et al*., 2024). H_2_S-based treatments are regarded as an economic and reliable approach to improve tolerance to a wide range of abiotic stresses (Choudhary *et al*., 2024). The use of NO donors, particularly sodium nitroprusside, as seed priming agents resulted in increased germinability, vigour, and stress tolerance, and similar results were obtained when NO donors were also applied in combination with other signalling molecules (Ciacka *et al*., 2022; Fejes *et al*., 2025). Based on these premises, the most challenging step will be to integrate current knowledge into a framework able to design strategies for a strong response to MFSC. However, there are still open questions to be addressed (Fig. 2). Such an effort will benefit from in-depth investigations at the cellular and molecular level since the overall picture is still poorly defined.

## DNA damage response and the seed response to multi-factorial stress combination: open questions

**Fig. 3.**
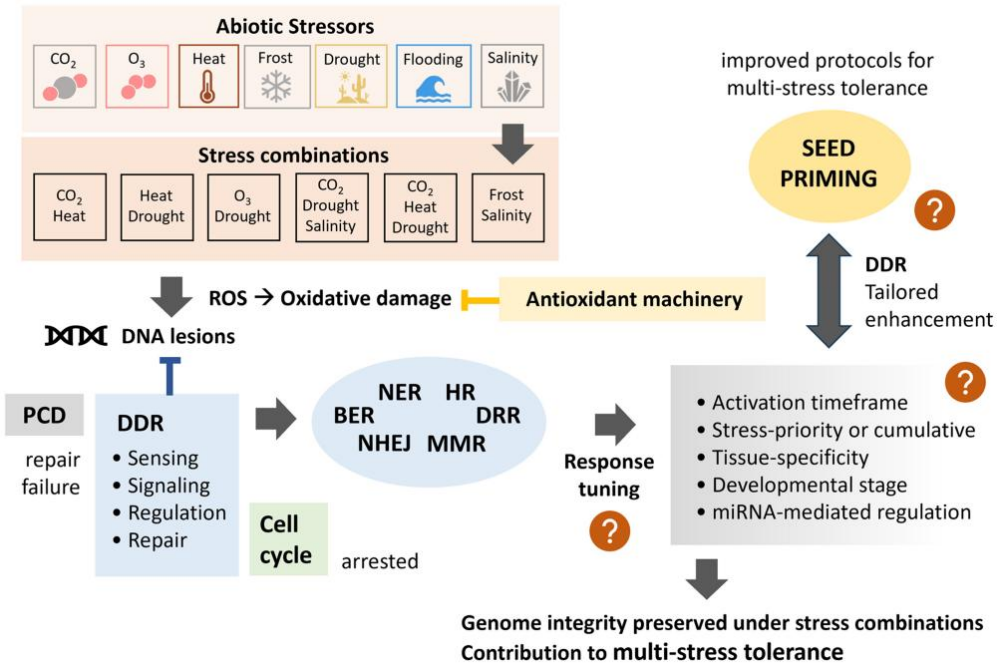
Schematic representation of the impact of combined abiotic stresses on the seed repair response. DDR ensures genome maintenance through the cascade of molecular events leading to the recruitment of the different components involved in DNA repair at the damaged sites. However, in the case of severe genotoxic damage, PCD is activated. Cell cycle arrest is necessary to allow DNA repair. How do seeds manage DDR when they are exposed to combined climatic stressors? A fine-tuning of the DDR pathways and players may be necessary, and the dynamics of such responses should be investigated. The resulting knowledge will hopefully be translated into improved seed priming protocols able to specifically boost the main DDR components crucial for the multi-stress resilience. BER, base excision repair; DDR, DNA damage response; DRR, direct reversal repair; HR, homologous recombination; MMR, mismatch repair; NER, nucleotide excision repair; NHEJ, non-homologous end joining; PCD, programmed cell death; ROS, reactive oxygen species.

Seed priming can undoubtedly contribute to advances in both basic and applied research aimed at developing novel climate-ready crop varieties with multi-stress tolerance, since it triggers specific responses for genome maintenance. Looking into these dynamics will help in defining to what extent DDR contributes to multiple stress tolerance and how the different DDR players are engaged. As DDR is a highly conserved mechanism, spanning across the plant and animal kingdoms, and considering the common output of different types of abiotic stresses, it is evident that such protective mechanisms are evoked under MFSC. A more intriguing question relates to the way in which DDR players can be utilized in seeds challenged with MFSC. As shown in Fig. 3, two possible options are envisaged: (i) a cumulative usage of the same DNA repair pathway or DDR players that is ‘amplified’ as many times as the number of occurring combined stresses; and/or (ii) the predominance of one or more specific DNA repair pathways or DDR players that are boosted, with respect to the other components. Additional research questions are raised when considering the spatio-temporal dynamics of such ‘cumulative’ or ‘specific’ usage of DDR components made by seeds (Fig. 3). Indeed, each option reflects different regulatory mechanisms at the level of gene expression; for example, in the ‘cumulative’ response, the expression of DDR genes could be increased, providing overall transcript levels significantly higher than those observed in response to each separate stress. Such a boost in gene up-regulation may require faster regulatory mechanisms, if the higher toxic ROS wave resulting from the concomitant exposure to multiple stresses increases in short time frames. Given the complexity of the regulatory network, this perspective should also be considered for the many post-translational processes that control protein stability and catalytic activity. It is possible that the miRNA world could contribute to shape such a landscape (Fig. 3). This class of highly conserved small non-coding RNAs that negatively regulate gene expression post-transcriptionally play key roles in relation to DDR in the context of seed germination (Tondepu *et al*., 2024).

## Conclusion

Understanding and predicting the effects of multiple stressors on crop performance and, more specifically, the way in which seeds cope with MFSC is currently one of the most important challenges for researchers. The knowledge of the molecular and physiological responses orchestrated to cope separately with different types of abiotic stresses, such as drought, salinity, and heat, is expanding at a faster rate compared with the state of the art dedicated to MFSC, and such a gap must be urgently filled. Several critical steps must be addressed. The huge biodiversity found in crop species relies on a range of highly conserved physiological, biochemical, and molecular mechanisms that, however, often display genotype-dependent profiles. This variability is typically observed in the response to seed priming since the current protocols used by seed operators and farmers, from field up to industrial scale, show genotype- and seed lot-dependent heterogeneous responses. To narrow down such empirical effects, researchers should work on a wider range of germplasm collections, looking at the phenotype level for distinctive features in terms of developmental speeds and stress tolerance levels. Other challenging aspects relate to the way in which the balance between fitness and optimization of stress tolerance occurs. Is it possible to quantify the costs of multi-stress adaptation in terms of metabolic energy demand and fitness? Concerted cross-disciplinary efforts are needed to explore this issue through the design of dedicated experimental systems, modelling, and sampling on a range of spatial and temporal scales (Orr *et al*., 2020). When considering the issues of genome integrity, multiple stressor research is still in its infancy and it will significantly benefit from exchange of knowledge and cross-fertilization of ideas. Multiple official data sources and worldwide organizations have provided evidence that climate change, particularly extreme meteorological events, represents a pressing challenge for the seed sector and ultimately for agricultural yield and food security. Research conducted so far has identified putative players and metabolic pathways to generate MFSC-tolerant plants. The modulation of the DDR throughout seed priming has the potential to bring innovation to the seed sector, contributing to more resource-efficient, resilient, and sustainable farming. The challenge now is to stimulate multi-player joint efforts to ensure that the knowledge generated is used to deploy innovative solutions (processes, products, or services) capable of addressing the real needs of the seed private sector and that it is efficiently adopted by farmers, to boost socio-economic growth and ultimately secure food and feed for all.
